# Adolescents’ academic achievement and meaning in life: the role of self-concept clarity

**DOI:** 10.3389/fpsyg.2025.1596061

**Published:** 2025-06-19

**Authors:** Fang Liu, Hanqi Li, Haoyang Sun, Peng Wang, Mengyuan Qin

**Affiliations:** ^1^Department and Institute of Psychology, Ningbo University, Ningbo, Zhejiang Province, China; ^2^Heqin Preschool Education College, Ningbo Childhood Education College, Ningbo, Zhejiang Province, China

**Keywords:** academic achievement, search for meaning, presence of meaning, self-concept, meaning-making model, adolescent well-being

## Abstract

Sense of meaning in life is closely related to adolescents’ well-being. While prior research has largely examined the effects of individual traits and environmental influences, adolescents’ personal experiences (e.g., academic achievement) also play a crucial role. This study investigates the relationship between adolescents’ academic achievement and their sense of meaning in life, with a focus on the moderating role of self-concept clarity. Drawing on the meaning-making model, we hypothesized that academic achievement would positively predict both the presence of and search for meaning in life, and that self-concept clarity would moderate these relationships. A total of 1,321 junior high school students (50.9% female; M_age_ = 12.39, SD_age_ = 0.52) from Henan Province, China, participated in the study. Participants completed a self-report measure of academic achievement, along with standardized scales assessing meaning in life and self-concept clarity. Results indicated that academic achievement was positively correlated with both presence of meaning and search for meaning. Self-concept clarity moderated the relationship between academic achievement and meaning in life, adolescents with higher self-concept clarity and higher academic achievement reported greater presence of meaning and search for meaning. The findings demonstrate that academic achievement significantly contributes to adolescents’ sense of meaning in life, and critically, self-concept clarity acts as a moderating variable that amplifies this positive association. Interventions targeting self-concept development could be a promising avenue for enhancing adolescents’ meaning in life and overall well-being.

## 1 Introduction

Meaning in life refers to an individual’s perception of purpose and significance in their existence, serving as a psychological resource that fosters positive emotional states and enhances overall well-being. This construct comprises two dimensions: the presence of meaning (one’s current sense of purpose) and the search for meaning (the pursuit of purpose), both crucial for psychological functioning (Chu and Lowery, 2024; Steger et al., 2008). Cultivating meaning in life is particularly vital during adolescence, as it acts as a protective factor promoting healthy developmental outcomes (King and Hicks, 2021).

The development of meaning in life is influenced by individual trait factors (e.g., personality characteristics and emotional patterns) and environmental factors (e.g., early adverse experiences), while it also being shaped by stage-specific individual’s experiences and significant life events within developmental context (Chen S. et al., 2024; Chen Z. L. et al., 2024; Edwards et al., 2025; Kwok et al., 2024; Park and Baumeister, 2017; Yuan et al., 2024). For adolescents, academic life occupies a substantial portion of their daily lives, and their individuals’ experiences related to academic life may influence their sense of meaning in life. Academic performance, as a key school outcome, represents one of the most important aspects of school experience and has been demonstrated to correlate with adolescents’ life satisfaction and subjective well-being (Mitchell et al., 2022; Clarke et al., 2025). Adolescents’ personal experiences, such as friendship and trauma, are closely associated with their sense of meaning in life (Chen et al., 2021; O'Rourke et al., 2019). In this similar vein, academic achievement, as a key adolescents’ experience, may also relate to meaning in life. Prior research on meaning in life has predominantly focused on individual traits and environmental factors (Edwards et al., 2025; Kwok et al., 2024). However, little is known about the relationship between academic achievement and meaning in life. Therefore, the current study aims to examine such the relationship.

According to the meaning-making model, individuals need to compare, evaluate, and judge the sense of meaning derived from situational stimuli with their existing beliefs (Steger, 2012). For adolescents, learning constitutes the primary source for evaluating their situational meaning, making learning-related evaluations highly significant for developing and pursuing their sense of meaning (Park, 2010). When adolescents face academic success or failure, they evaluate these events against their existing self-beliefs (e.g., “I am a competent student”). If a discrepancy arises (e.g., failing despite effort), they engage in meaning-making: either assimilating the experience (e.g., attributing failure to temporary factors) or accommodating their beliefs (e.g., revising self-perceptions or goals). To our knowledge, few studies have directly examined the relationship between academic achievement and meaning in life. However, we can draw on related research. A substantial body of research has investigated links between adolescent academic achievement and their life satisfaction and subjective well-being (Bailey and Phillips, 2016; Bücker et al., 2018; Clarke et al., 2025). For example, a cross-sectional study of 607 teenagers found adolescents’ academic achievement positively correlated with their subjective well-being (Clarke et al., 2025). Longitudinal studies have also demonstrated that adolescents’ GPA significantly predicts their subjective well-being 1 year later (Steinmayr et al., 2016). Given the close relationship between subjective well-being and meaning in life, including conceptual overlap (Li et al., 2021), it is reasonable to hypothesize that adolescent academic achievement positively relates to their meaning in life.

However, not all adolescents with high academic achievement experience a stronger sense of meaning in life. Due to individual differences in meaning-making processes, the impact of low academic achievement may be weak or insignificant for some individuals, as adolescents with poor academic performance can derive meaning from alternative sources. A meta-analysis confirmed a small-to-moderate correlation between academic achievement and subjective well-being (Bücker et al., 2018). These findings suggest the existence of potential moderating variables in the relationship between academic achievement and its psychological outcomes. One such factor is self-concept clarity, which refers to the extent to which beliefs about self (e.g., personality traits, values, and beliefs) are clearly and confidently defined, internally consistent, and stable, is an essential foundation for individuals to live a meaningful life (Chen S. et al., 2024; Chen Z. L. et al., 2024). According to the meaning-making model, when individuals encounter new information, they process it by either assimilating it (fitting it into existing beliefs) or accommodating (adjusting their beliefs to fit the new information). If the new information relates to their identity, their response depends on how firmly their existing self-beliefs are held. Research showed that people with low self-concept clarity were more likely to change their self-descriptions, whereas those with high self-concept clarity tended to maintain stable self-concept (Akın et al., 2015). In other words, individuals with clear self-concepts not only just assimilate new information, but also actively use situational cues to strengthen their existing beliefs. This suggests that students with clear self-concepts may derive stronger meaning from academic success, as they can more effectively integrate achievement-related experiences into their stable self-views.

Self-concept encompasses an individual’s cognitive and evaluative perceptions of themselves and their relationship with the environment. This construct develops progressively through socialization processes and reflects the degree of self-awareness development (Chu and Lowery, 2024; Markus and Wurf, 1987). Research has shown that self-concept is closely related to adolescents’ mental health and life satisfaction (Chen S. et al., 2024; Chen Z. L. et al., 2024; Crone et al., 2022). Longitudinal research in the United States revealed that an increase in self-concept clarity corresponded to an increase in meaning in life (Shin et al., 2016). And there is study shown that self-concept clarity is negatively associated with anxiety and depression (Tokunaga and Horiuchi, 2012). As a protective factor for adolescent development, self-concept plays a crucial role in regulating the relationship between individual performance and subjective feelings (Sticca et al., 2020). A cross-sectional and longitudinal study among Chinese adolescents showed that individuals with higher self-concept clarity might have higher levels of grit resulting in higher life satisfaction (Chen S. et al., 2024; Chen Z. L. et al., 2024). Self-concept clarity may influence how adolescents process and interpret environmental information. When facing negative life events or setbacks (e.g., low academic achievement), those with low self-concept clarity may experience greater difficulty coping with these challenges, reducing their ability to derive meaning from such experiences. Conversely, adolescents with clearer self-concept can better allocate cognitive resources toward seeking meaningful pursuits beyond academic performance.

Guided by the meaning-making model (Park, 2010; Steger, 2012), this study examines the relationship between adolescents’ academic achievement and their sense of meaning in life (comprising presence of meaning and search for meaning), with a focus on the moderating role of self-concept clarity. By integrating self-concept clarity into the meaning-making framework, this study advances understanding of how academic achievement fosters a meaning in life during adolescence—a critical period for identity formation (Erikson and Erikson, 1998). Therefore, we propose the following hypotheses in this study:

*H1*: Academic achievement is positively correlated with meaning in life.

*H2*: Self-concept clarity moderates the relationship between academic achievement and meaning in life, the positive relationship will be stronger for adolescents with higher self-concept clarity.

## 2 Methods

### 2.1 Participants

In this study, seventh and eighth grade adolescents were recruited as participants. First, two junior high schools were selected from Henan Province, China. Then, using a cluster sampling approach (with grades as strata), we selected five classes per grade within each school as sampling clusters, all students in the selected classes were invited to participate in the study by their head teachers. A pencil-and-paper survey was distributed to students of randomly selected classes in each school. A total of 1,360 students participated in the study. After excluding 39 invalid questionnaires (including straight-lining and non-differentiation response patterns, as well as incomplete questionnaires), the final analysis included 1,321 participants (50.9% female) aged between 11 and 15 years (*M* = 12.39, SD = 0.52). All participants were right-handed, had normal or corrected-to-normal vision, and provided written informed consent. The study design was approved by the Human Research Ethics Committee of Ningbo University. 89.4% of the participants reported no issues with their parental marital status. 60.6% of the participants reported that their parents’ educational attainment was at the elementary school level, 23.1% at the junior high school level, and 16.3% at the high school level or above. The subject family financial situation scores ranged from 1 to 10 (*M* = 5.42, SD = 1.27), higher scores indicate better family financial situation.

### 2.2 Materials

#### 2.2.1 Academic achievement

Academic achievement was assessed using a single-item measure: “How would you rate your academic achievement.” Our use of a single-item measure aligns with prior research demonstrating strong validity for academic self-perceptions (Leung and Xu, 2013). Participants were asked to respond on a 5-point Likert scale (1 = “Very poor” to 5 = “Very good”), with higher scores indicating better academic achievement.

#### 2.2.2 Meaning in life questionnaire

The Chinese revised version of the Meaning in Life Questionnaire (MLQ) was adapted from the Chinese version developed by Liu and Gan in their study (Wang, 2013). The scale consists of two subscales: the Presence of Meaning (MLQ-P) and the Search for Meaning (MLQ-S). Each subscale contains 5 items, rated on a 7-point Likert scale (1 = “Completely disagree “to 7 = “Completely agree “). Higher scores indicate a greater sense of meaning in life. MLQ has shown satisfactory validity and reliability in a Chinese cultural context (Wang, 2013). In this study, the questionnaire demonstrated good Cronbach’s *α* values, including the total score (0.81), Presence of Meaning (0.81), and Search for Meaning (0.81).

#### 2.2.3 Self-concept clarity scale

The Chinese revised version of the Self-Concept Clarity Scale (SCCS) was adapted from the Chinese version developed by Niu et al. (2016) in their study. The scale consists of 12 items designed to measure the clarity and consistency of an individual’s self-concept (e.g., “My beliefs about myself often conflict with each other”). The items are rated on a 4-point Likert scale (1 = “Strongly disagree” to 4 = “Strongly agree”). Higher scores indicate a higher level of self-concept clarity. The scale has shown satisfactory validity and reliability in a Chinese cultural context, Cronbach’s α was 0.81, (Niu et al., 2016). In this study, the Cronbach’s α was 0.79.

### 2.3 Procedure

Ethical approval was granted by Ethics Committees at the first author’s University. In addition, informed consent was obtained from both students and their teachers before completing the measures. All participants were told that their privacy would be protected and they were free to withdraw from the study at any time. The anonymity of the study was also emphasized before data collection. The first author distributed the questionnaires to all students with the help of the teacher in the classroom. Trained research assistants were present during the data collection process to offer assistance and ensure that participants had a comprehensive understanding of the questionnaire items. Trained research assistants gave clear and standardized instructions, and also checked if participants understood by asking them to explain some questions in their own words. During the data collection process, they looked out for any confusion and answered questions whenever needed. Participants completed the meaning in life questionnaires, self-concept clarity scale, and demographics variables in a quiet classroom.

### 2.4 Data analysis

Data were processed and analyzed using SPSS version 27.0 and R studio. Listwise deletion estimate was used to handle missing data (Enders, 2022). Following listwise deletion a total of 4.1% of data were missing within the analyzed sample. MCAR test indicated that the data was missing completely at random (*p* = 0.49). First, data were analyzed in SPSS 27.0. Demographic characteristics were analyzed with descriptive statistics. Pearson correlation analysis was used to evaluate the relationships between bivariate variables, and a linear regression was performed to make predictions about the variables of interest. Last, moderation analyses were conducted using the bruceR package in R Studio with the significance of 95% confidence intervals. According to Cheung’s suggestions, we employed 5,000 bootstrap resamples—a number shown to provide stable confidence interval estimates for indirect effects in simulation studies (Cheung et al., 2023).

## 3 Results

First, descriptive analysis as well as Pearson’s coefficients were depicted in Table 1. Academic achievement was positively correlated with presence of meaning (*r* = 0.17, *p* < 0.001), search for meaning (*r* = 0.13, *p* < 0.001) and self-concept clarity (*r* = 0.06, *p* = 0.033), self-concept clarity was positively correlated with presence of meaning (*r* = 0.31, *p* < 0.001) and was negatively correlated with search for meaning (*r* = −0.18, *p* < 0.001).

**Table 1:** Descriptive statistics and correlation coefficient matrix (*N* = 1,321).

Variables	*M*	SD	1	2	3	4	5	6
Presence of meaning	23.158	6.103	–					
2. Search for meaning	24.043	5.782	0.306^***^ (<0.001)	–				
3. Self-concept clarity	32.661	6.154	0.308^***^ (<0.001)	−0.183^***^ (<0.001)	–			
4. Academic achievement	3.200	0.988	0.171^***^ (<0.001)	0.133^***^ (<0.001)	0.059^*^ (0.033)	–		
5. Gender	1.490	0.500	0.078^**^ (0.005)	0.001 (0.961)	0.098^***^ (<0.001)	−0.028 (0.311)	–	
6. Age	12.390	0.520	0.015 (0.600)	−0.026 (0.345)	−0.053 (0.057)	−0.029 (0.293)	0.092^***^ (<0.001)	–

^*^p < 0.05, ^**^p < 0.01, ^***^p < 0.001. Gender is coded as a categorical variable (male = 0, female = 1). The numbers in parentheses indicate the *p*-values corresponding to the correlation coefficients.

Second, a lineal regression in the prediction of presence of meaning and search for meaning was carried out. The model was statistically significant for presence of meaning, as described as follows: *F* (5,1256) = 35.85; MSE = 1160.60; *R*^2^ = 0.13; *p* < 0.001. Moreover, the model was also statistically significant for search for meaning: *F* (5,1256) = 14.44; MSE = 456.776; *R*^2^ = 0.05; *p* < 0.001. Coefficients are depicted in Table 2.

**Table 2:** Linear regression coefficients on the meaning in life scores.

Model	Predictor	B	SE	*β*	*t*	*p*
Presence of meaning	Intercept	23.142	0.158	−0.004	146.505	0.000^***^
	Gender	0.001	0.001	0.016	0.603	0.547
	Age	0.001	0.001	0.010	0.363	0.717
	SES	−0.000	−0.000	−0.003	−0.130	0.897
	Academic achievement	0.925	0.160	0.150	5.769	0.000^***^
	Self-concept clarity	0.294	0.026	0.297	11.391	0.000^***^
	AA × SCC	0.064	0.026	0.064	2.501	0.013^*^
Search for meaning	Intercept	24.023	0.155	−0.006	155.465	0.000
	Gender	0.001	0.001	0.014	0.504	0.614
	Age	−0.000	0.002	−0.001	−0.044	0.965
	SES	0.002	0.001	0.046	1.718	0.086
	Academic achievement	0.805	0.157	0.138	5.130	0.000^***^
	Self-concept clarity	−0.186	0.025	−0.199	−7.369	0.000^***^
	AA × SCC	0.063	0.025	0.067	2.515	0.012^*^

AA is the abbreviation for academic achievement, and SCC is the abbreviation for self-concept clarity. Standardized coefficients are presented. ^***^p < 0.001, ^*^p < 0.05.

**Figure 1:**
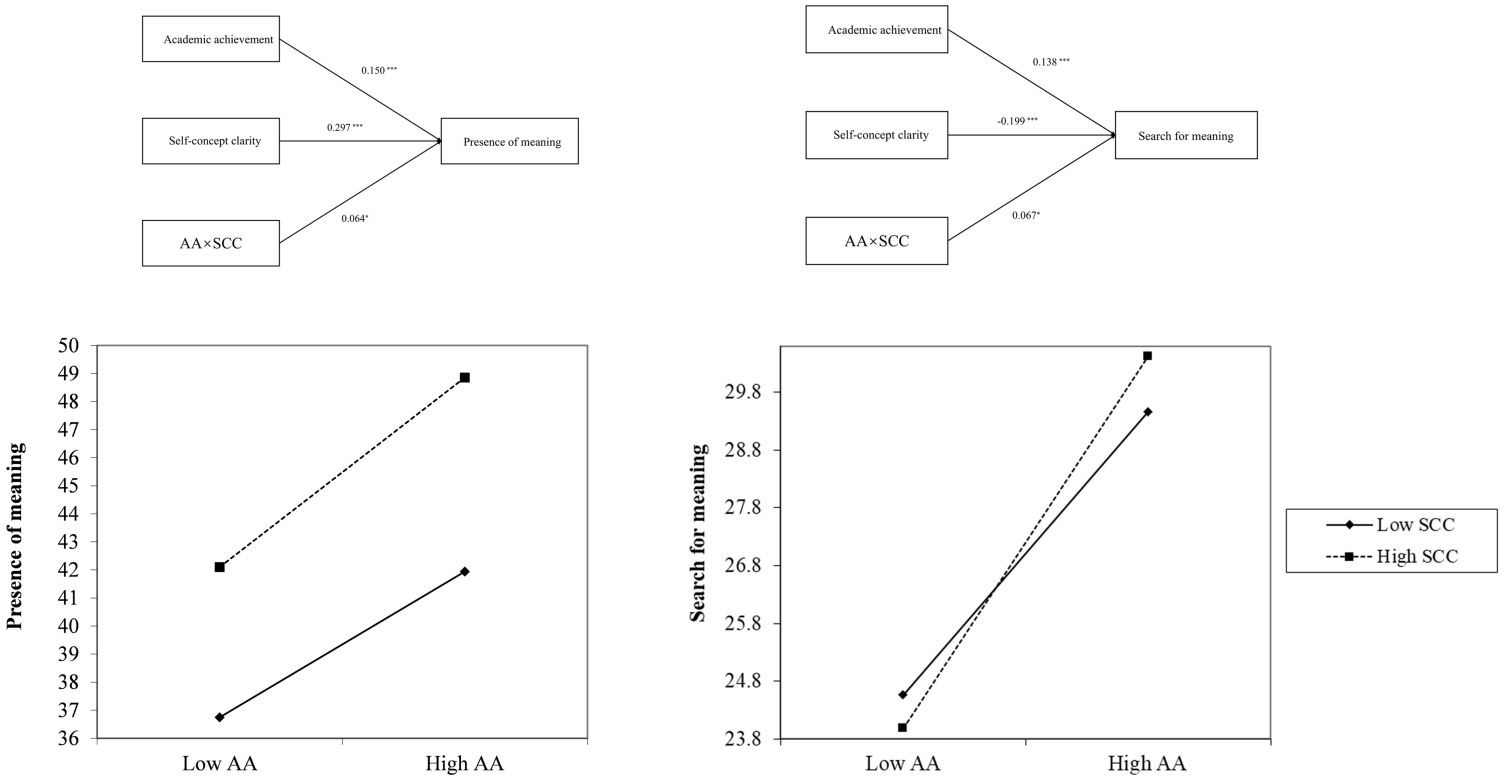
Moderation model for academic achievement and presence of meaning and search for meaning across self-concept clarity. AA is the abbreviation for academic achievement, and SCC is the abbreviation for self-concept clarity. Standardized coefficients are presented. ^***^p < 0.001, ^*^p < 0.05.

**Figure 2:**
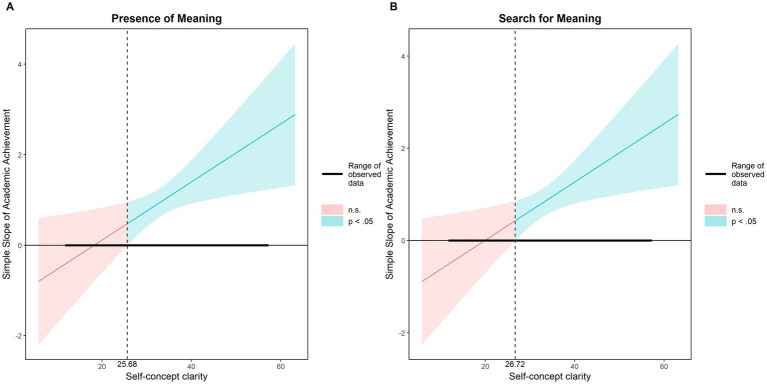
Johnson-Neyman plot of Self-Concept Clarity’s Moderating Effect. SCC, Self-Concept Clarity. For Presence of Meaning, **(A)** the effect of Academic Achievement is significant when SCC > 25.68. For Search for Meaning, **(B)** the effect is significant when SCC > 26.72. Observed SCC range: 12.00 to 57.00 for both analyses.

Last, a moderation model on self-concept clarity over the relationship between academic achievement and presence of meaning and search for meaning was carried out. Moreover, Figure 1 depicts the proposed models and interactions. Self-concept clarity did moderate the relationship between academic achievement and the presence of meaning, and it also moderated the relationship between academic achievement and the search for meaning. In the first case, the moderation model on presence of meaning was statistically significant: *F* (6,1309) = 30.51; MSE = 32.72; *R*^2^ = 0.12; *p* < 0.001. The R^2^ increase due to the interaction depicted the following values: *R*^2^ = 0.004; *p* < 0.05. In the second case, the moderation model on search for meaning was also statistically significant: *F* (6,1309) = 14.07; MSE = 31.31; *R*^2^ = 0.06; *p* < 0.001. The R^2^ increase due to the interaction depicted the following values: *R*^2^ = 0.005; *p* < 0.05. Coefficients depicted in Figure 1, as well as the interaction, also reached the statistical level. Additionally, we have used Johnson-Neyman analysis to present the confidence intervals in Figure 2. The conditional effect of academic achievement on presence of meaning and search for meaning at values of the self-concept clarity is described in Table 3.

**Table 3:** Conditional effect of academic achievement on presence of meaning and search for meaning at values of the self-concept clarity.

Moderator level	Outcome variable	Effect	SE	*t*	*p*	LLCI	ULCI
Low (−1 SD) self	presence of meaning	0.086	0.037	2.322	0.020^*^	0.013	0.158
Mean self		0.150	0.026	5.769	<0.001^***^	0.099	0.201
high (+1 SD) self		0.214	0.036	5.925	<0.001^***^	0.143	0.285
Low (−1 SD) self	search for meaning	0.071	0.038	1.862	0.063	−0.004	0.146
Mean self		0.138	0.027	5.130	<0.001^***^	0.085	0.190
high (+1 SD) self		0.205	0.037	5.476	<0.001^***^	0.131	0.278

Effects, standard error (SE), statistical significance, and lower and upper (LLCI and ULCI) level.

## 4 Discussion

This study investigates the moderating effect of self-concept clarity on the relationship between adolescents’ academic achievement and their meaning in life. As expected, adolescent academic achievement positively predicts meaning in life. Moreover, compared to adolescents with low academic achievement, those with high achievement demonstrated significantly higher levels of presence of meaning under conditions of high self-concept clarity. Similarly, adolescents with high academic achievement exhibited significantly higher levels of search for meaning under high self-concept clarity, relative to those with low achievement.

Adolescent academic achievement showed a significant association with their meaning in life. These findings support the meaning-making model, proposing that adolescents compare and evaluate situational meaning with their general meaning in life. For teenagers in their student years, academic performance is an important indicator to their meaning in life. Academic success provides adolescents with a sense of competence and purpose, reinforcing their meaning in life (Steger, 2012). Compared with adolescents with low academic achievement, adolescents with high academic achievement were more likely to align their evaluation of situational meaning with their general meaning, resulting in higher levels of presence of meaning (Park, 2010; Steger, 2012). These results align with extant literature. For instance, Lu et al. (2024), in their large-scale meta-analysis incorporating studies from 29 countries, documented inverse associations between depressive symptoms and academic performance. A study conducted among Spanish adolescents further indicates that academic achievement positively correlated enhanced life satisfaction (Ayllón-Salas and Fernández-Martín, 2024). Analogously, high academic achievers exhibit greater engagement in search for meaning. This pattern corroborates existing evidence that elevated presence of meaning predicts stronger search for meaning, facilitating personal development (Lutz et al., 2023). Notably, a 14-day diary study revealed that both positive and negative achievement events correlated with daily fluctuations in meaning among adolescent students (Machell et al., 2015).

The results further identified self-concept clarity as a significant moderator. Moderation analyses revealed that heightened self-concept clarity significantly amplifies the presence of meaning. Self-concept enabling adolescents across diverse racial/ethnic groups with well-defined self-concept to better recognize their strengths and cultivate meaning in life (Chu and Lowery, 2024). This amplification effect implies that clear self-concept acts help adolescents strengthen their personal significance. With such self-concept clarity, high achievers may integrate their academic experiences into their self-identity more effectively, leading to stronger perceptions of meaning. A clearly articulated self-identity fosters self-certainty, which subsequently enhances perceptions of coherence, purpose and significance - culminating in stronger presence of meaning, particularly when coupled with high academic achievement (Chu and Lowery, 2024). Our research is also supported by previous studies. For instance, Hong et al. (2022) found that self-concept clarity buffered the negative effects of depression among nursing students, with meaning in life playing a mediating role. Similarly, Liu et al. (2023) demonstrated that self-concept clarity strengthened the positive association between life meaning and learning engagement, suggesting that individuals with higher self-concept clarity derive greater motivational benefits from a sense of purpose. Furthermore, self-concept clarity emerged as equally influential in motivating search for meaning. High-achieving adolescents demonstrated greater propensity for search for meaning, particularly when possessing well-developed self-concept clarity. Academic success may stimulate deeper existential curiosity when students have a stable self-identity. In contrast, those with low self-concept clarity might avoid meaning exploration due to uncertainty. A three-day diary study utilizing a Chinese sample demonstrated that search for meaning predominantly emerges alongside well-differentiated self-concept (Chen S. et al., 2024; Chen Z. L. et al., 2024). Clear self-understanding enhances the effective pursuit of life objectives. Consequently, academically successful adolescents can allocate greater resources to meaning exploration, whereas those struggling academically often remain preoccupied with scholastic demands, constraining extra-academic meaning pursuits. Erikson’s developmental theory emphasizes adolescence as the pivotal stage for identity formation, wherein youth actively construct self-identity. Unresolved identity formation during this period often necessitates prolonged exploration to achieve identity consolidation (Erikson and Erikson, 1998). Additionally, we observed a weak correlation between academic achievement and self-concept clarity (*r* = 0.06), the correlation aligns with theoretical and methodological expectations. Theoretically, adolescence involves fluid self-concept development, wherein academic success does not invariably consolidate identity clarity. Methodologically, as a hypothesized moderator, self-concept clarity should not exhibit a strong linear association with achievement. We also observed a negative correlation between self-concept clarity and search for meaning. This correlation suggests that adolescents with clear self-concept exhibit less meaning exploration. When self-knowledge is clear, individuals experience greater presence of meaning and consequently exhibit reduced motivation to seek external sources of purpose.

The current findings carry important implications for promoting adolescent life education and positive youth development. First, adolescents should be encouraged to develop robust self-concept alongside their academic pursuits. Previous research has demonstrated that mindfulness-based interventions (MBIs) can significantly enhance adolescents’ self-concept by improving self-awareness and reducing negative self-evaluation (Gómez-Odriozola and Calvete, 2021). For practical implementation, schools could integrate brief mindfulness exercises (e.g., 10-min guided breathing or body-scan sessions) into daily routines. Second, academic performance should not serve as the sole metric for evaluating adolescent development. Empirical research demonstrates that adolescents’ soft skills developed through extracurricular activities (e.g., adaptability, curiosity, perseverance) is positively associated with life satisfaction and self-regulated learning which are of great significance to students’ development (Feraco et al., 2023). Parents and educators should guide adolescents to maintain balanced perspectives on academic performance, cultivate extracurricular interests and engage in self-exploration activities—all of which may collectively enhance meaning in life (Shoshani and Steinmetz, 2014).

Several study limitations warrant consideration. First, the cross-sectional design precludes causal inferences, necessitating cautious interpretation of the observed associations. Future research should employ multi-wave longitudinal designs to examine temporal dynamics in these relationships. Second, reliance on self-reported academic achievement may introduce measurement bias. Moreover, a single-item measurement may be difficult to provide a comprehensive academic performance, and caution is needed when explaining the practical significance. Future investigations should incorporate objective achievement metrics (e.g., standardized test scores, GPA). Third, in the regression model analyzing “search for meaning” the *R*^2^ value is only 0.05, indicating that the model has limited explanatory power regarding the dependent variable. This outcome may reflect the multidimensional nature of the complex psychological phenomenon associated with “search for meaning” as well as the potential influence of unmeasured variables not accounted for in the current model. Future research could enhance the model’s explanatory power by refining the research design, increasing the sample size, and utilizing more precise measurement instruments. Forth, while our study identified statistically significant moderation effects, the observed Δ*R*^2^ values were small. This is consistent with typical effect sizes in psychological research examining complex interactions (Asal et al., 2025; Sageer et al., 2024), but requires cautious interpretation regarding practical applications. Future research should examine whether these moderation effects show stronger impacts in targeted subgroups (e.g., adolescents undergoing identity transitions). Finally, the exclusive focus on junior high school students precludes examination of potential age-related effects. Future work should encompass broader developmental periods to elucidate how these associations vary across adolescence.

## 5 Conclusion

This study employed a moderated model to examine the relationship between adolescents’ academic achievement and meaning in life, and the critical moderating role of self-concept clarity in this association. Results demonstrated that adolescents’ academic achievement is positively related to their meaning in life. Compared to low academic achievement adolescents, adolescents with high academic achievement demonstrated higher levels of both presence of meaning and search for meaning under conditions of high self-concept clarity. Self-concept clarity amplified the beneficial effects of academic achievement on meaning in life, particularly for adolescents with higher academic performance. These findings suggest that self-concept clarity may be a critical psychological resource in translating academic success into a sense of meaning in life, interventions targeting self-concept development could be a promising avenue for enhancing adolescents’ meaning in life and overall well-being in future research and educational practice.

## Data Availability

The raw data supporting the conclusions of this article will be made available by the authors, without undue reservation.
